# Specialty preferences and factors affecting the choices of postgraduate specialty among undergraduate medical students

**DOI:** 10.12669/pjms.38.6.5571

**Published:** 2022

**Authors:** Elsadig Yousif Mohamed

**Affiliations:** 1 Dr. Elsadig Yousif Mohamed, MD. Department of Community Medicine College of Medicine, Majmaah University Al-Majmaah 11952, Saudi Arabia

**Keywords:** Specialty preferences, Postgraduate Specialty, Medical students

## Abstract

**Objectives:**

To study specialties preference of the students at the College of Medicine, Majmaah University, Saudi Arabia; and to determine the factors that affect the choice of their postgraduate specialty.

**Methods:**

A cross-sectional study was conducted among 138 undergraduate medical students at the College of Medicine, Majmaah University, Saudi Arabia for the current academic year 2021-2022. Data were collected using a questionnaire. Data were analyzed using SPSS version 24, and a Chi-square test was utilized to compare qualitative data.

**Results:**

Internal medicine is the first choice for 16 (19.8%) students. General surgery and family medicine were selected by 15 (18.5%) and 12 (14.8%) students, respectively. Sixty-seven (82.7%) students relied on their selection of a good future guaranteed by specialty, and 48 (49.3%) selected challenging specialty; 64 (79.0%) were motivated by a practicing doctor for their future specialty selection and 54 (66.7%) by their families.

**Conclusions:**

Most preferred specialty for medical students is internal medicine followed by general surgery. Both genders preferred internal medicine as their future specialty. Some important specialties such as public health and basic medical sciences were not selected as a future specialty by any student. The most stated reason behind specialty selection is the provision for a good future. Most students are motivated by a practicing doctor to select a postgraduate specialty.

## INTRODUCTION

National policies on distribution of postgraduate opportunities in medical colleges is determined by estimated projections for the need of medical specialties in the future. Selection of medical specialty is an important requirement in health planning for human resources, which has acquired increasing attention in the last decades.^1^,^2^ The tendency toward the selection of different medical specialties can largely influence the future landscape of the physician workforce in a healthcare system.^3^ Failure to adopt the right strategies to plan and adjust the medical specialties selection may lead to undesirable consequences for health systems, one of which is the imbalance between various specialties.^4^ This inadequate future planning may lead to a lack of health services in future specialties in the rural areas, while the primary health services will take the most part of it.^5^-^8^

The choice of a future career in medical practice can be a difficult task for medical students as there are many factors to consider.^8^,^9^ Previous studies have showed that students are generally inclined toward clinical specialties, and students in the clinical phase of their study tend to decide on a future specialty compared to students in the pre-clinical phase.^10^

Most students with a particular specialty are selected based on monetary criteria. Prior studies have showed that students fluent in English and skilled in computers are unlikely to be undecided in the choice of their future careers. However, other studies found that the main reasons behind specialization selection were primary interest in the specialty,^7^,^11^,^12^ job satisfaction^7^, personality type^13^, academic and educational determinants^10^, along with cultural and social values.^7^,^14^,^15^

In Saudi Arabia, the policies encourage Saudi nationals to pursue an early career pathway in the medical field to ensure a well-functioning and specialized healthcare system. Among Saudi students, lifestyle and social characteristics determine career path selection.^6^ Internal medicine, family medicine, general surgery, pediatrics, and emergency medicine were the most preferred specialties.^15^ Furthermore, the most preferred specialties among medical students were surgery followed by internal medicine, according to a previous study.^1^

The current study aimed to determine the specialties preference of the students at the College of Medicine, Majmaah University, Saudi Arabia; determine the factors that affect the choice of their postgraduate specialty, and verify whether there is a relationship between future career choice and sociodemographic characteristics.

## METHODS

A cross-sectional and community-based study was conducted among undergraduate medical students at the College of Medicine, Majmaah University, Saudi Arabia. The registered students of both genders for the current academic year 2021-2022 formed the sample frame of this study. The sample was collected by simple random sampling using the table of random selection. The final sample size was 138.

Data were collected by a pre-tested questionnaire after obtaining the ethics approval from Majmaah University IRB (HA-01-R-088). Informed consent was obtained from all participants. The reliability of our study’s questionnaire was Cronbach’s alpha = 0.732. The questionnaire included socio-demographic characteristics and questions related to specialty preference, reasons behind the selection, and individuals whose career path choices were affected. Cronbach’s alpha was used to ensure the questionnaire’s reliability. The questionnaire was tested on medical students at the College of Dentistry, Majmaah University. Difficult and ambiguous questions were revised. Data analysis was performed using SPSS version 24. The Mean ± SD was reported for age. The Chi-square test was utilized to compare qualitative data, and a p-value of less than 0.05 was considered significant.

## RESULTS

Cociodemographic characteristics of the students are presented in Table-I. The mean age of the students was 22.0±2.58 years, sixty-eight percent being males and most of them were singles (96.4%). Most students were in the fourth year of education (36.2%). Students with high and low academic performance (CGPA) were 37.7% and 17.4% respectively. Most students (65, 49.2%) have a family income of more than 20,000 SR/ month. The level of father and mother education of most students was college and above (44.9%) and (52.9%) respectively.

**Table I:** Sociodemographic characteristics of the students (n=138).

Sociodemographic characteristics	No.	%
***Gender:***		
Male	95	68.8
Female	43	31.2
***Marital status:***		
Single	133	96.4
Married	5	3.6
***Educational Level:***		
2^nd^ year	24	17.4
3^rd^ year	30	21.8
4^th^ year	50	36.2
5^th^ year	15	10.8
6^th^ year	19	13.8
***Cumulative Grade Point Average (CGPA)***		
Low (less than 3.5)	24	17.4
Average (3.5-3.9)	40	29.0
High (4.0- 4.4)	52	37.7
V. high (4.5-5.0)	22	15.9
***Family monthly income/SR:***		
Less than 5000	9	6.5
5000-10000	30	21.8
10001- 20000	31	22.5
More than 20000	65	49.2
***Father’s education level:***		
Primary	23	16.7
Intermediate	19	13.7
Secondary	34	24.7
College and above	62	44.9
***Mother’s education level:***		
Primary	27	19.6
Intermediate	20	14.5
Secondary	18	13.0
College and above	73	52.9

Most students (58.7%) expressed their future specialty. Internal medicine was the first choice for 19.8% students. General surgery and family medicine were selected by 18.5% and 14.8% students respectively as listed in Table-II.

**Table II:** Choice of specialty (n=81).

Specialty	No.	%
Internal medicine	16	19.8
General Surgery	15	18.5
Family medicine	12	14.8
Orthopedics	5	6.2
Neurology	6	6.2
Obstetrics and Gynecology	5	6.2
Emergency Medicine	5	6.2
Pediatrics	4	4.9
Anesthesia	3	3.7
Cardiac Surgery	3	3.7
Radiology	3	3.7
Others (Ophthalmology, Dermatology, Pathology, Urology)	4	6.2

The reasons behind specialty selection are shown in Table-III. Eighty two point seven percent students stated that the selected specialty will provide a good future and 49.3% stated that the selected specialty is challenging. The most influencing person in specialty selection was a practicing doctor (79.0%) followed by the family (66.7%).

**Table III:** Sources and reasons behind specialty selection (n=81)

Reasons behind specialty selection	No.	%
Will provide a good future	67	82.7
Challenging specialty	48	59.3
Interesting and will provide a higher income	42	51.9
Impact on patients’ quality of life	40	49.3
Intellectually stimulating	38	46.9
High demand in the job market	38	46.9
Prestigious job	34	42.0
Looking at the future	31	38.3
Diverse problems are seen in practice	23	28.3

*Sources behind specialty selection*	*No.*	*%*

Practicing doctor	64	79.0
Family	54	66.7
Colleague	39	48.1
Friend	38	46.9
Faculty member	35	43.2
Relative	16	19.8

The relationship between specialty selection and gender are presented in Table-IV. Forty eight point four percent of the male students preferred internal medicine for their future specialty, followed by general surgery 27 (42.2%). Seventy-point six percent of the female students preferred internal medicine, followed by general surgery 4 (23.5%). The relationship between specialty selection and gender was significant (p=0.006).

**Table IV:** Relation between specialty selection and gender.

Specialty	Gender		Total	P

	Male No. (%)	Female No. (%)		
General Surgery	27 (42.2%)	4 (23.5%)	31 (38.2%)	
Internal Medicine	31 (48.4%)	12 (70.6%)	43 (53.1%)	0.006
Others (anesthesia and radiology, BMS)	6 (9.4%)	1 (5.9%)	7 (8.7%)	

Total	64 (100.0%)	17 (100.0%)	81 (100%)	

## DISCUSSION

One hundred and eighty-three undergraduate students responded to the questionnaire provided during the study. Eighty-one students (58.7%) expressed their future preference of specialty. This result is higher than the finding of a study conducted in Kuwait, in which only 37.2% of the students expressed their future preferred specialty.^10^ The most preferred future specialty for the students was internal medicine (21.1%), followed by general surgery (19.7%). This finding is consistent with other studies conducted elsewhere.^9^,^16^ General Surgery, ENT, ophthalmology, and internal medicine were the most preferred specialties in a study conducted by Abdulghani et al,^17^ while Al-Fouzan, in her study conducted in Kuwait, found that pediatrics, general surgery, and cardiology was the most desired specialties.^6^ In a study conducted in Saudi Arabia, general surgery and internal medicine was the most desired specialties.^15^ Another study found that internal medicine was the preferred specialty, followed by family medicine and general surgery.^18^

In the current study, basic medical sciences and public health disciplines were not selected as a future specialty by any student. This finding is consistent with that of a study conducted in China, Sri Lanka, India, and Nepal. None of the students in these countries preferred pediatrics^2^. Abdulghani found that community medicine, forensic medicine, and obstetrics/gynecology were the least selected specialties even for female medical students.^17^

In our study, male and female students selected internal medicine as their first choice (48.8% vs. 70.6%), followed by general surgery (42.2% vs. 23.5%, p=0.006). In a study conducted in Saudi Arabia, men preferred pediatrics and emergency medicine, while women preferred family medicine.^14^ This finding contradicts the study of Kakkar et al who found that the first choice for females was obstetrics and gynecology.^16^

Practicing doctors followed by family were the most influential sources concerning specialty selection.^12^,^15^ In a study conducted among final year medical students in Nigeria, Okunlola found that the most important factors influencing postgraduate selection are personal interest and family influence.^19^

In the current study, most students (82.7%) stated that the provision of a good future is a major reason behind specialty selection, followed by the presence of challenging specialty (59.3%). This finding is consistent with other studies.^16^,^18^-^21^ Studies conducted elsewhere found that prestige, respect, and status are the most important factors behind specialty selection.^12^,^22^ Researchers also stated that economic concerns were the first reason behind career selection.^19^,^23^,^24^ Chawla et al found that passion for the subject and financial gain were the main reasons behind specialty selection^3^. The most stated reasons behind specialty preference in studies conducted in Kuwait included looking for a good treatment outcome for patients, the presence of interesting cases, and the ability to maintain long-term relationships in the discipline^6^. A study conducted among female students in Taif University, Saudi Arabia stated that the first reason behind specialty selection was career development.^25^

### Limitations of the study

This study was conducted at Majmaah University, the results will be of more importance if conducted among all universities in Saudi Arabia.

## CONCLUSION

The most preferred specialty for medical students in this study was internal medicine, general surgery, and family medicine. Both genders preferred internal medicine as their future specialty, followed by general surgery. The reasons behind specialty selection included the provision of a good future, being involved in a challenging specialty, and the interest and impact of specialty in patients’ quality of life. The influential people behind specialty selection for most of the students included a practicing doctor, family, and colleagues.

For health planners, the study recommends starting specialty in disciplines including basic medical sciences and public health early in the program. Furthermore, students should be directed toward these specialties early during their university study period. Practicing doctors are advised to be part of the campaigns for career selection to help students select a suitable specialty. Planners are advised to provide incentives to direct medical students toward specialties that are typically avoided as future careers.
